# Exploring electroencephalographic chronic pain biomarkers: a mega-analysis

**DOI:** 10.1016/j.ebiom.2025.105955

**Published:** 2025-09-30

**Authors:** Felix S. Bott, Paul Theo Zebhauser, Vanessa D. Hohn, Özgün Turgut, Elisabeth S. May, Laura Tiemann, Cristina Gil Ávila, Henrik Heitmann, Moritz M. Nickel, Melissa A. Day, Divya B. Adhia, Yoni K. Ashar, Tor D. Wager, Yelena Granovsky, David Yarnitsky, Mark P. Jensen, Joachim Gross, Markus Ploner

**Affiliations:** a Department of Neurology, School of Medicine and Health, Technical University of Munich (TUM), Munich, Germany; b TUM-Neuroimaging Center, School of Medicine and Health, TUM, Munich, Germany; c Center for Interdisciplinary Pain Medicine, School of Medicine and Health, TUM, Munich, Germany; d Institute for AI and Informatics in Medicine, TUM, Munich, Germany; e School of Psychology, University of Queensland, Brisbane, QLD, Australia; f Department of Surgical Sciences, Otago Medical School, University of Otago, Dunedin, New Zealand; g Department of Psychiatry, Weill Cornell Medical College, New York City, NY, USA; h Department of Psychology and Neuroscience, University of Colorado, Boulder, USA; i Laboratory of Clinical Neurophysiology, Bruce Rappaport Faculty of Medicine, Technion Israel Institute of Technology, Haifa, Israel; j Department of Rehabilitation Medicine, University of Washington, Seattle, WA, USA; k Institute for Biomagnetism and Biosignalanalysis, University of Münster, Münster, Germany

**Keywords:** Electroencephalography, Biomarkers, Chronic pain, Large-scale brain networks, Replicability

## Abstract

**Background:**

Chronic pain is associated with alterations in brain function, offering promising avenues for advancing diagnostic and therapeutic strategies. In particular, these alterations may serve as brain-based biomarkers to support diagnosis, guide treatment decisions and monitor clinical courses of chronic pain.

**Methods:**

Motivated by this potential, this study analysed associations between chronic pain and changes of large-scale brain network function using resting-state electroencephalography (EEG) from 614 individuals with chronic pain, collected by research groups from Australia, Germany, Israel, New Zealand, and the US.

**Findings:**

Employing a discovery-replication approach, we found limited replicability of associations between pain intensity and brain network connectivity. However, a mega-analysis combining all datasets revealed robust associations between pain intensity and large-scale brain network connectivity at theta frequencies and including the limbic network. Additionally, multivariate analyses identified connectivity patterns spanning theta, alpha, and beta frequencies with strong evidence for associations with pain intensity. Variations and ablations of model features yielded deeper insights into the relative importance of distinct electrophysiological brain features in assessing chronic pain.

**Interpretation:**

Our findings highlight challenges and provide guidance for developing EEG-based, scalable, and affordable biomarkers of chronic pain.

**Funding:**

This project was funded by the 10.13039/501100001659Deutsche Forschungsgemeinschaft and the 10.13039/501100005713Technical University of Munich.

**Research in context**

**Evidence before this study**

Chronic pain is a major healthcare challenge and developing biomarkers that objectively assess pain is a crucial step toward improving its diagnosis and treatment. Brain-based biomarkers are particularly promising, as they can capture the complex interplay of biological, psychological, and social factors underlying chronic pain. Electroencephalography (EEG) is a cost-effective and widely accessible tool for assessing brain function. Previous studies have linked chronic pain to standard EEG features, such as frequency-specific power and peak alpha frequency. However, a systematic review has highlighted inconsistencies in these findings, likely due to small sample sizes and methodological differences. Brain connectivity, which reflects interactions between different brain regions, has been proposed as a more informative marker of chronic pain. While some EEG studies have explored connectivity patterns, findings have been limited by small sample sizes and a lack of replication across independent datasets.

**Added value of this study**

This study presents the largest EEG dataset of individuals with chronic pain to date, incorporating data from 614 participants acquired globally by research groups from Australia, Germany, Israel, New Zealand, and the US. Our preregistered, state-of-the-art EEG analyses provide three key insights: First, our findings challenge previous findings. We found no replicable evidence linking chronic pain to standard EEG features commonly reported in earlier studies. Second, the results identify novel EEG biomarker candidates. We found strong evidence for associations between chronic pain and connectivity-based EEG features spanning theta, alpha, and beta frequencies. Third, our observations inform biomarker development. By dissecting our connectivity models, we identified concrete avenues for the development of EEG-based biomarkers of chronic pain.

**Implications of all the available evidence**

Building on previous research on brain connectivity in neuropsychiatric disorders, this study highlights the potential of EEG-based connectivity measures as biomarkers for chronic pain. By integrating heterogeneous datasets from multiple sources, we also demonstrate key challenges and propose strategies for ensuring the replicability of EEG biomarkers in chronic pain and other brain disorders. Ultimately, scalable and validated EEG biomarkers could support more objective assessments and personalised treatment approaches.

## Introduction

Chronic pain is a multi-faceted and debilitating condition that significantly burdens individuals and society.^1^^,^^2^ Converging lines of evidence indicate that the brain plays a central role in the development and maintenance of chronic pain.^2^^,^^3^ Thus, a better understanding of the brain's role in chronic pain might help develop new approaches for diagnosing, predicting, and treating chronic pain. In particular, brain-based biomarkers could enhance clinical care by informing treatment decisions, tracking disease progression, and serving as objective endpoints in clinical trials. As a step toward identifying such biomarkers, the present study investigates how alterations in brain function, captured with electroencephalography (EEG), relate to chronic pain.

Several standard features of resting-state EEG, including frequency band-specific signal power and peak alpha frequency, have been proposed as biomarker candidates for chronic pain.^4^ However, the reported associations were inconsistent, likely due to small sample sizes and variability in analysis approaches across studies.^4^ Brain connectivity is another important EEG feature that might be particularly informative about chronic pain. This is suggested by findings revealing that contextual influences on pain relate to inter-regional connectivity rather than local brain activity.^5^ Since contextual factors play a crucial role in chronic pain, measures of interregional brain connectivity might capture chronic pain particularly well. Moreover, both functional magnetic resonance imaging and EEG studies have demonstrated the informative value of connectivity analyses for assessing neuropsychiatric disorders closely associated with chronic pain, such as depression and post-traumatic stress disorder.^6^, ^7^, ^8^, ^9^, ^10^

In this study, we specifically investigated connectivity among large-scale brain networks. Large-scale brain networks, also known as intrinsic brain networks, are spatially extended networks of brain regions that share similar functional properties.^11^^,^^12^ Previous fMRI studies have linked alterations in the function of these networks to different brain disorders, including chronic pain.^13^, ^14^, ^15^, ^16^, ^17^, ^18^ These large-scale brain networks were identified using fMRI. However, their functional organisation likely represents a fundamental feature of brain activity^19^^,^^20^ that is also accessible by other assessments of brain function, including EEG. As EEG is broadly available, cost-efficient, and potentially mobile, EEG-based biomarkers have an exceptionally high potential for translation into clinical use.

**Fig. 1:**
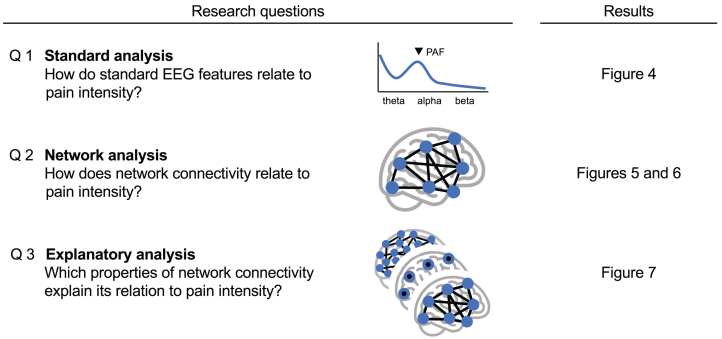
**Research questions.** We used EEG data from eight independent studies involving people with chronic pain to investigate how brain connectivity relates to pain intensity.

Here, we investigated the connectivity across large-scale brain networks in the largest EEG database of individuals with chronic pain to date. Moreover, we used this unique database to re-investigate the relationship between standard EEG features and chronic pain intensity. We assessed the replicability and consistency of findings based on eight independent datasets (n = 614), including two datasets recorded by our research group in Germany and six datasets recorded by research groups in Israel, New Zealand, the US, and Australia. We specifically addressed three research questions (Fig. 1). First, we assessed how pain intensity relates to standard EEG features previously linked to chronic pain (*standard analysis*). Second, we assessed how pain intensity relates to connectivity between large-scale brain networks (*network analysis*). Third, to understand which components of our network model drove the associations with pain intensity, we compared it to several alternative models that used different network properties as features (*explanatory analysis*).

## Methods

### Overview

In this study, we investigated how standard EEG features and connectivity between large-scale brain networks relate to pain intensity in people with chronic pain. To this end, we analysed eight resting-state EEG datasets of people with chronic pain (total n = 614, Table 1). The participants had different types of chronic pain, with chronic back pain being the most frequent type (n = 405). Further types of chronic pain included chronic widespread pain, joint pain, and neuropathic pain. Data from participants of all genders were analysed jointly. Gender-disaggregated participant information is provided in Table S1. The datasets were recorded at various sites with different devices and setups but were centrally collected and analysed at one site. To assess the relationship between EEG features and pain intensity and the replicability of the findings, we pursued a discovery-replication approach. The discovery dataset consisted of a large sample recorded by the group in Munich (n = 119). The replication datasets were the other seven datasets (n = 47–123, total n = 495). In addition, we conducted mega-analyses that combined all eight datasets into a single joint dataset (n = 614).

**Table 1:** Overview of datasets.

Dataset name	N_192_/N_ALL_	N_CBP_	N_CWP_	N_JP_	N_NP_	N_OTHER_	#sens
**Discovery set**							
*Set_Munich1*	119/127	74	13	12	20	0	64
**Replication sets**							
*Set_Munich2*	63/88	21	2	11	17	12	32
*Set_Brisbane*	60/61	60	0	0	0	0	64
*Set_Otago1*	57/57	57	0	0	0	0	64
*Set_Otago2*	77/77	77	0	0	0	0	64
*Set_Boulder*	68/68	68	0	0	0	0	19
*Set_Haifa*	47/112	0	0	0	47	0	64
*Set_Seattle*	123/148	48	0	0	0	75	128
**Total**	**614/738**	**405**	**15**	**23**	**84**	**87**	

The study included eight datasets, each comprising resting-state EEG and metadata of people with different types of chronic pain. To ensure accurate EEG feature estimates, we included only participants with a minimum of 192 clean EEG data epochs (see Methods section for details). The numbers N_192_ and N_ALL_ represent the counts of participants with at least 192 clean epochs and the total number of participants, respectively. Chronic back pain (CBP) was the most frequent type of chronic pain. Further types chronic pain included chronic widespread pain (CWP), joint pain (JP), neuropathic pain (NP), and miscellaneous types of chronic pain (OTHER). Visualisations of the distributions of the individuals' pain intensity ratings and age for each dataset are provided in Fig. S1.

We performed univariate analyses to assess associations between individual EEG features and pain intensity. In addition, we performed multivariate analyses to investigate associations between patterns of brain network connectivity and pain intensity. Univariate and multivariate analyses relied on Bayesian statistics, which allowed for interpreting both positive and negative findings. We interpreted Bayes factors (BF) > 1, >3, and >10 (<1, <1/3, <1/10) as anecdotal, moderate, and strong evidence in favour of (or against) an effect.

We first analysed the discovery dataset and then used the replication sets to quantify the replicability of the effects. *Replicability* of effects in independent data was quantified by a BF reflecting the strength of evidence for (or against) a correlation, in the same direction as in the discovery set, between an EEG feature and pain intensity in the pooled replication sets. This BF was interpreted as anecdotal, moderate, or strong evidence for (against) replicability, as defined above. Finally, we performed mega-analyses^21^^,^^22^ on the joint dataset comprising the discovery and all replication sets. Mega-analyses resolve the hierarchy between discovery and replication sets and are more sensitive with respect to more subtle effects present across datasets.

The study was preregistered at osf.io (https://osf.io/qa68n).

### Ethics

This study involved secondary analysis of de-identified, anonymised data obtained from controlled-access repositories. No new data were collected, and there was no interaction with or intervention involving human participants. As such, this analysis does not constitute human subjects research and did not require additional ethical approval. All original studies from which data were derived had received appropriate ethical approval from local ethics committees (IRB numbers are provided in the dataset descriptions below) and in accordance with the Declaration of Helsinki, as documented in their respective publications.^23^, ^24^, ^25^, ^26^, ^27^, ^28^, ^29^, ^30^, ^31^

### Datasets and dataset harmonisation

We based our analyses on eyes-closed resting-state EEG recordings in people with chronic pain. We used two EEG datasets from our research group (*Set_Munich1*, *Set_Munich2*) and identified and acquired six external EEG datasets (*Set_Brisbane*, *Set_Otago1*, *Set_Otago2*, *Set_Boulder*, *Set_Haifa*, and *Set_Seattle*).

#### Data acquisition strategy

To acquire external datasets, we approached research groups from Australia, Brazil, China, Denmark, Germany, Israel, Italy, New Zealand, Spain, and the US in a structured data acquisition campaign. We identified candidate datasets using a two-step strategy. First, we relied on a previously published, peer-reviewed systematic review of EEG studies in chronic pain populations, covering publications up to the end of 2021.^4^ Second, we manually screened studies listed in PubMed and published in 2022 to ensure inclusion of more recent data. While we deem the resulting list of studies representative of the literature, we acknowledge that a broader database search (e.g., including Scopus) might have yielded additional eligible datasets. To select external datasets, we applied three criteria: number of EEG sensors ≥32; publication date ≥2013; and number of participants ≥20, balancing expected data quality and resource constraints. After filtering, 18 studies (some sharing the same dataset) remained. Corresponding authors were contacted for data sharing and we sent a standardised follow-up message to non-responders after several weeks. Four external groups contributed datasets. Additionally, we included one dataset recorded with fewer than 32 channels (Set_Boulder) because it had been obtained early in the project and incorporating it entailed no additional effort. Thus, in total, we had eight independent resting-state EEG datasets at our disposal (overview in Table 1, detailed description below). Note that, although we applied systematic inclusion criteria and contacted all eligible studies, only a subset of groups agreed to share data. The others either did not respond, no longer had access to the data, or wished to conduct further analyses before sharing the data. While we believe the risk of selection bias is limited, given that data were shared before analysis and our hypotheses differed from those in the original studies, we acknowledge that the included datasets may not fully represent the broader literature, and this may impact generalisability.

**Fig. 2:**
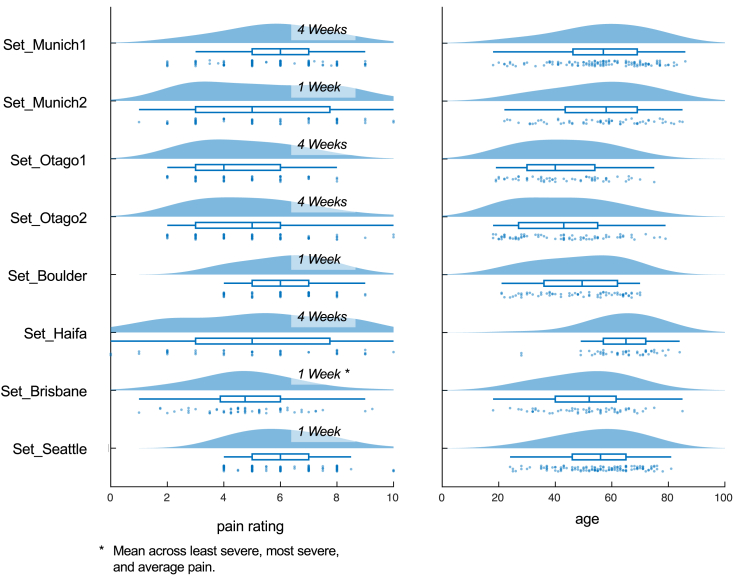
**Pain intensity and age distributions.** For the distributions of pain intensity, it is additionally specified for each dataset to which time period prior to the assessment the ratings refer.
• **age:** Age of the participant at the time of the recording.
• **diagnosis:** Identifier for category of pain diagnosis: Chronic back pain, not chronic back pain (i.e., chronic widespread pain, neuropathic pain, joint pain, or chronic pain of other etiologies).

In addition to EEG recordings, we utilised the following clinical and demographic information:

• **pain intensity:** Average pain over a period of one to four weeks prior to assessment, rated on an 11-point numerical rating scale ranging from no pain (0) to worst imaginable pain (10). The time periods to which the ratings refer in the individual studies can be inferred from Fig. 2. Note that these ratings may not be the same as those used during participant selection in the individual studies.

#### Data harmonisation

As the data originate from multiple sites, we implemented a rigorous multi-step harmonisation workflow. First, raw files received in diverse formats were converted to the Brain Imaging Data Structure (BIDS) specification. Second, we performed fully automated preprocessing with the preprocessing module of the DISCOVER-EEG pipeline^32^ (see below). To ensure comparability, we retained only the first 192 artifact-free 2 s epochs from each resting-state, eyes-closed recording. From these epochs, we extracted diverse EEG features, including standard spectral features and network connectivity measures (see below). Remaining site effects were mitigated using ComBat, a batch-effect correction tool frequently used in multicentre neuroimaging studies^33^, ^34^, ^35^ and recently validated specifically for resting-state EEG features.^36^ ComBat employs an empirical Bayesian framework that improves parameter estimation in smaller samples. It has demonstrated superior performance compared to alternative strategies such as raw feature residuals or including site as a regressor to the predictive model.^34^^,^^37^^,^^38^ In this study, ComBat was applied to mitigate site-related variability while preserving variance associated with the biologically meaningful factor of age, except when age was the dependent variable. In line with our preregistered approach, we removed site-related variance from the dependent variables (pain intensity and age) by computing within-site z-scores. Moreover, as several measures of brain function have been linked to age,^32^^,^^39^^,^^40^ we also regressed out age from both independent and dependent variables in all analyses, except when age itself was the dependent variable. In line with our preregistered protocol, we did not include additional confounders beyond age. While incorporating further confounders could further support the robustness of our findings, the necessary data were not consistently available across datasets. Moreover, compared to age, we found less prior evidence supporting their potential confounding influence on pain ratings or measures of brain function. As explained in more detail below, in multivariate analyses, ComBat-harmonisation, z-scoring, and confounder removal were done in a manner to prevent leakage of information from test to training sets in the employed cross-validation procedures.

#### Datasets

*Set_Munich1* (IRB number: 5493/12) is composed of multiple datasets that have previously been recorded in our research group to investigate brain dysfunction in people with chronic pain.^23^, ^24^, ^25^ These datasets have been used in combination to assess measures of brain activity, brain connectivity,^24^ brain dynamics^41^ (i.e., microstate analyses^42^), and excitation/inhibition^43^ (i.e., 1-over-f analyses^44^) cross-sectionally in people with chronic pain and healthy controls, as well as to assess measures of brain activity and brain connectivity longitudinally in people with chronic pain.^25^ Here, we included only baseline recordings of the longitudinal dataset. In all studies of *Set_Munich1*, inclusion criteria consisted of a clinical diagnosis of chronic pain, with pain persisting for at least six months and with an average pain intensity of at least four (two in the case of the longitudinal dataset) on an 11-point numerical rating scale (NRS) ranging from zero (no pain) to ten (worst imaginable pain) during the four weeks prior to the assessment. People with severe diseases other than chronic pain or those taking regular benzodiazepine medication were excluded. In total, *Set_Munich1* comprised data from 127 people with chronic pain. After preprocessing, eight participants had to be excluded due to not meeting the minimum epoch number requirement. The analysed cohort (n = 119) consisted of 74 people with chronic back pain (CBP), 13 people with chronic widespread pain (CWP), 20 people with neuropathic pain (NP), and 12 people with joint pain (JP). All datasets were recorded using a passive electrode EEG system with 64 channels (Easycap, Herrsching, Germany) and BrainAmp MR plus amplifier (Brain Products, Munich, Germany). Previous analyses of *Set_Munich1* did not yield any evidence which could have biased the analyses of the present study.

*Set_Munich2* (IRB number: 6/22 S-KH) resulted from a study assessing pain medication effects on EEG-based measures of brain function.^26^ In total, datasets of 88 people with chronic pain were available, 25 of whom had to be excluded due to not meeting the minimum epoch number requirement after preprocessing. Among the remaining n = 63 participants, there were 21 with CBP, two with CWP, 17 with NP, 11 with JP, and 12 with pain of other etiologies (OTHER). To record EEG in this study, a 32-channel system with active dry electrodes (CGX-Quick32r, CGX-systems, San Diego, US) was used.

*Set_Brisbane* (IRB number: 2015000568) resulted from a study that investigated the effectiveness of several non-pharmacological, 8-week interventions for the treatment of CBP in 69 people with chronic pain.^27^ To be eligible for the study, participants had to report pain in the lower back area for more than three months with an average pain intensity of at least a 4 on an 11-point NRS ranging from 0 to 10 in the four-week period prior to assessment. Participants with severe psychiatric comorbidities were excluded from the study. After preprocessing, data from 60 participants recorded prior to interventions could be included in the present study. EEG recordings were obtained using an ANT Neuro EEGO sports system (Medical Imaging Solutions GmbH, Berlin, Germany) with 64 active scalp electrodes (Waveguard cap).

*Set_Otago1* (IRB number: 20/CEN/60) was recorded as part of a study investigating the efficacy of infra-slow neurofeedback training as a treatment for chronic low back pain in 60 participants.^28^ Eligibility criteria were analogous to those stated for *Set_Brisbane*. For the analyses presented here, we used baseline data from 57 participants meeting the minimum epoch number requirement. EEG recordings were obtained using a 64-electrode system with SynAmps-RT amplifier (Compumeics-Neuroscan, Abbotford, Australia).

*Set_Otago2* (IRB numbers: 20/NTB/67, 2023 EXP 17953) was recorded as part of studies investigating the effects of neurofeedback and transcranial electrical stimulation on chronic back pain. The dataset was collected by the same researchers, using equivalent recording conditions and eligibility criteria as those used for the recording of *Set_Otago1*.

*Set_Boulder* (IRB number: 16-0544) is a dataset for which, to date, no analyses have been published. It was recorded in the context of a larger study investigating the efficacy of pain reprocessing therapy for the treatment of chronic back pain.^29^ Eligibility criteria were very similar to those described for datasets *Set_Brisbane*, *Set_Otago1*, and *Set_Otago2*. *Set_Boulder* comprises data from 68 people with CBP which were recorded using a 19-channel EEG system (Evoke system, evoke neuroscience, New York, USA). No participant had to be excluded due to not meeting the minimum epoch number requirement.

*Set_Haifa* (IRB number: 0052-15-RMB) comprises recordings from 112 people with painful diabetic polyneuropathy, i.e., belonging to the NP category. In the present study, we used data from 47 participants which fulfilled the minimum epoch number requirement after preprocessing. In the original study, these data were used to train a machine learning model to distinguish participants with painful from those with non-painful diabetic polyneuropathy.^30^ This study was part of the larger DOLORisk^45^ initiative aiming to identify risk factors for the development and maintenance of neuropathic pain. Inclusion criteria defined by this initiative were, e.g., having a diagnosis of Type 1 or Type 2 diabetes and having a clinical diagnosis of peripheral neuropathy or symptoms highly suggestive thereof. For EEG recordings, a 64-channel system with active electrodes was used (ActiCHamp, Brain Products, Munich, Germany).

*Set_Seattle* (IRB number: 43605 G) stems from a study which investigated the relative effects of hypnotic cognitive therapy, standard cognitive therapy, hypnosis focused on pain reduction, and pain education in adults with a variety of chronic pain conditions.^31^ Here, we focus on the first 5 min of resting state EEG recordings acquired prior to the interventions. In total, data from 148 participants were available, 123 of which met our minimum epoch number requirement. Among these, 48 had CBP, the remainder of participants had chronic pain secondary to multiple sclerosis (n = 45), spinal cord injury (n = 21), or muscular dystrophy (n = 9). The inclusion criteria of the original study required potential participants to report an average pain intensity of at least four on an 11-point NRS, ranging from 0 to 10, in the past week. Moreover, participants had to report pain on at least 50% of the days in the past four weeks. Potential participants were excluded from the study if they had previously received psychological treatment or any other form of treatment akin to the treatments investigated in the study. EEG recordings were conducted using a 128-channel hydrocell net connected to a GES 300 high-density EEG acquisition system (magnetism EGI, Eugene, USA). Here, we only included data from the 116 sensors which were located in regions covered by the head model employed for source reconstruction.

##### Preprocessing

For our analyses we used exclusively eyes-closed EEG recordings as these have been shown to give rise to more robust results.^46^ We first selected the earliest 192 clean 2 s epochs (with 50% overlap) of each individual's EEG recording. This was done to ensure that we consistently used neural data from early phases of the recording. Moreover, by using a fixed number of epochs, we excluded any sample size biases in EEG feature estimates. We opted for a fixed number of 192 clean epochs as it represented a good trade-off between the quality of feature estimates (increasing with the number of epochs) and the number of included participants (decreasing with the number of epochs). EEG data from all sites were preprocessed in a uniform manner using an automatic preprocessing pipeline, which was initially proposed by^47^ and adapted in^32^ for the use of resting-state recordings. This pipeline represents a concatenation of several established functions from the Matlab-based EEGLAB toolbox.^48^ It comprises the following steps: Downsampling to 250 Hz, line noise removal, bad channel rejection, re-referencing to average reference, independent component analysis and automated rejection of independent components labelled by a machine learning classifier as “muscle” or “eye”,^49^ bad segment rejection, and epoching. In all preprocessing functions, we used the default parameter settings.

### Evaluating brain measures

In this study, we primarily assessed source-level brain connectivity and activity at theta (4–8 Hz), alpha (8–<13 Hz), and beta (13–30 Hz) frequencies. The connectivity between two brain structures (brain networks, regions, or locations) was defined as the amplitude envelope correlation (AEC)^50^ between those brain structures' representative signals (see below). We chose AEC deliberately for several reasons. First, AEC is a widely validated and frequently used method for assessing functional connectivity in resting-state EEG, particularly in clinical populations.^10^^,^^51^^,^^52^ Second, our study is embedded in a large-scale brain network framework originally developed using resting-state fMRI,^11^^,^^53^ where functional connectivity is defined by correlations in slow signal fluctuations. AEC captures co-fluctuations in amplitude envelopes over time, making it conceptually closer to fMRI-based connectivity than phase synchronisation measures. This alignment was critical for interpreting our results within established neuroimaging network models. Third, the AEC implementation we used incorporates symmetric orthogonalisation of signals,^50^ a computational step explicitly designed to suppress spurious zero-lag correlations arising from common sources or sensor leakage. The activity of a brain structure was defined as the log-transformed, absolute variance of signals (i.e., power) within that brain structure which could be explained by that brain structure's representative signal.

In addition to network-related features, we computed the following standard, sensor-level EEG-features using the DISCOVER-EEG pipeline^32^: global, absolute signal power at theta, alpha, and beta frequencies and two versions of the peak alpha frequency (local maximum and centre of gravity of the power spectral density in the alpha band). Herein, the global signal power in a given band was determined by averaging the power spectral density within that band and across all sensors.

#### Source reconstruction

To reconstruct source-level brain activity, we employed Linearly Constrained Minimum Variance (LCMV) beamformers^54^ implemented in the Matlab-based FieldTrip toolbox.^55^ Frequency-specific array-gain LCMV spatial filters for theta, alpha, and beta frequencies were constructed based on a lead field and a frequency-specific covariance matrix. The lead field was computed by a boundary element approximation of the solution to the bioelectromagnetic forward problem for a realistically shaped, three-shell head model. The covariance matrix was estimated for each individual frequency band based on the band-pass filtered EEG epochs. To ensure a robust inversion of the covariance matrix, we employed Tikhonov regularisation as implemented in FieldTrip with a regularisation parameter value of 5% of the average sensor power. The fixed orientation of the lead field for every source location was chosen to maximise the variance of the spatial filter output. Source-level signals were then obtained by applying the frequency-specific LCMV operator to the band-pass filtered sensor-level time series. Spatial filters were computed for source locations corresponding to the centroids of brain parcels described by the Schaefer atlas.^56^ In all analyses except the explanatory network analysis, we used the variant of the Schafer atlas comprising 400 parcels. In extended network analyses, we used both the variant of the Schaefer atlas comprising 400, and the variant comprising 100 parcels. Subsequent analysis steps are based on the source-level signals.

#### Representative signals

To mitigate the influence of field spread, we computed brain connectivity using a representative signals approach. With this approach, information is aggregated on the level of individual brain structures (i.e., parcels, anatomical regions, or brain networks) by first computing signals representative of these brain structures.

The representative signal of a brain structure is often defined as the first principal component (PC) across all signals associated with that brain structure. A representative signal computed in this way constitutes the solution to an optimisation problem by maximising the explained variance across all signals associated with the given brain structure. Here, we refined this approach and added orthogonality constraints to this optimisation problem. Specifically, we defined the representative signal of a brain structure as the one that maximises the explained variance across all signals of that brain structure while constraining the explained variance of signals associated with other brain structures to zero. Two versions of our method exist:

• Pairwise orthogonalisation: For a given brain structure pair, an associated pair of orthogonal representative time series is estimated.
• Global orthogonalisation: For a given single brain structure, one representative time series is estimated that is orthogonal to a set of time series representing the activity outside of that brain structure. The number *N*_*c*_ of time series used to represent the activity outside of the brain structure of interest is a parameter of that method.

**Fig. 3:**
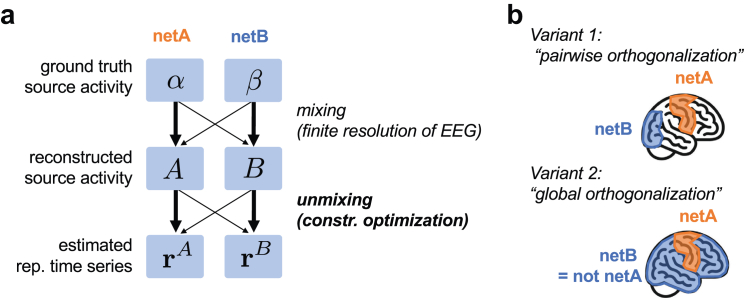
**Methods for estimating representative time series.** (a) Conceptual diagram of signal mixing due to imperfect source reconstruction (top half) and unmixing procedure (bottom half). (b) Illustration of two variants of the proposed method. Variant 1: For each network pair, one corresponding pair of orthogonal representative time series is determined. Variant 2: For each individual network, one representative time series is determined which is orthogonal to multiple (orthogonal) time series representing activity in all other networks.

More formally, consider networks netA and netB, for which we aim to obtain the representative time series $r^{A}$ and $r^{B}$, respectively. These representative time series should effectively capture the ground truth activity of netA and netB. In Fig. 3a, we denote the ground truth activity of netA and netB as α and β, respectively. However, due to the limited spatial resolution of EEG, we do not have direct access to this ground truth activity; instead, we only have access to a blurred estimate of it, which we denote as the source-reconstructed signals $A\in R^{k_{A}\times n}$ and $B\in R^{k_{B}\times n}$ with $k_{A}$ and $k_{B}$ denoting, respectively, the number of parcels belonging to networks netA and netB and n being the number of samples in the considered epoch. In the proposed approach, the representative time series $r^{A}$ and $r^{B}$ maximise the explained variance in A and B, respectively, while being orthogonal. By enforcing orthogonality between $r^{A}$ and $r^{B}$, we ensure that there is no shared portion of variance between them. Specifically, any variance in, say, $r^{A}$ cannot be explained by $r^{B}$. As $r^{B}$ is constructed to be representative of the activity in netB, this orthogonalisation reduces the contamination of $r^{A}$ by the activity in netB. Formally, this approach can be expressed as the constrained optimisation problem

$$\begin{array}{rr} \left(r_{1},r_{2}\right)= & \underset{r_{1},r_{2}}{\mathrm{argmax}}\left(\nu \left(r_{1},A\right)\;+\;\nu \left(r_{2},B\right)\right)\\ \mathrm{subject}\;\mathrm{to} & r_{i}=w_{i}^{⊤}\left[\begin{array}{c} A\\ B \end{array}\right]\;\forall \;i\in \left\{1,2\right\}\\ & r_{i}r_{j}^{⊤}=\delta_{ij}\;\forall \;i,j\in \left\{1,2\right\}\text{,} \end{array}$$

where δ is the Kronecker delta and, given that r has unit length and the mean of the rows of X equals zero, ν(r,X) is the fraction of the variance of matrix X explained by vector r, i.e.,

$$\begin{array}{r} \nu \left(r,X\right)=\frac{rX^{⊤}Xr^{⊤}}{tr\left(X^{⊤}X\right)}\text{.} \end{array}$$

For the sake of brevity in notation, the representative time series $r^{A}$ and $r^{B}$ have been denoted by $r_{1}$ and $r_{2}$, respectively, in the above equation. To solve this optimisation problem, a standard iterative optimisation algorithm is employed, which employs information from both the local gradient and Hessian.^57^ Note that, if we were to remove the orthogonality constraint $r_{1}r_{2}^{⊤}=0$, the representative time series would simply correspond to the first principal components of the data matrices A and B.

Expanding on this idea, we can consider a variant of the method where instead of obtaining a pair of time series for each pair of networks (*“pairwise orthogonalisation”*), we estimate a single time series for each network (*“global orthogonalisation”*). To achieve this, we designate netA as the network of interest and define netB as all regions in the brain not contained within netA (Fig. 3b). Since our focus is on obtaining a single time series only for netA, we can describe the activity of netB using multiple orthogonal components. The associated optimisation problem is

$$\begin{array}{rr} r_{1}= & \underset{r_{1},\ldots ,r_{N_{c}+1}}{\mathrm{argmax}}\left(\nu \left(r_{1},A\right)\;+\sum_{i=2}^{N_{c}+1}\nu \left(r_{i},B\right)\right)\\ \mathrm{subject}\;\mathrm{to}\; & r_{i}=w_{i}^{⊤}\left[\begin{array}{c} A\\ B \end{array}\right]\;\forall \;i\in \left\{1,\ldots ,N_{c}\;+\;1\right\}\\ & r_{i}r_{j}^{⊤}=\delta_{ij}\;\forall \;i,j\in \left\{1,...,N_{c}\;+\;1\right\}\text{,} \end{array}$$

where $N_{c}$ denotes the number of components used to describe the activity of netB and $r_{1}$ is the time series representative of netA.

Through simulation studies (see Supplementary Methods for details), we found that of the considered methods, the optimal choice is global orthogonalisation with *N*_*c*_ = 3. We, therefore, employed this variant as the standard approach for extracting representative signals. In explanatory network analyses, which explore different connectivity definitions, we additionally considered the first non-orthogonalised PCs as representative signals.

### Statistics

To examine associations between pain intensity and neural measures at the between-subject level, we performed both univariate and multivariate analyses. In univariate analyses, we computed correlations between pain intensity ratings and single neural measures. In multivariate analyses, we computed correlations between actual pain intensity ratings and predictions of pain intensity ratings generated by machine learning (ML) models. To assess correlations statistically, we computed Bayes factors (BFs) with default priors using the *correlationBF* function implemented in the BayesFactor package in R.^58^ To assess the potential influence of outliers, we also computed rank-based correlations (Spearman's ρ) and associated p-values (FDR-corrected). Note that, in multivariate analyses, we report correlation coefficients rather than the coefficient of determination (R^2^), which is also commonly used to assess prediction accuracy in machine-learning studies. This choice reflects our primary interest in the presence and strength of associations between predicted and observed pain ratings, rather than in the model's absolute predictive accuracy.

We integrated information from multiple datasets using a discovery + replication and a mega-analysis approach. In the discovery + replication approach, we first assessed bidirectional correlations in a designated discovery set (Set_Munich1). Depending on the direction of these correlations, we then tested for positive or negative correlations in the six replication sets, determining both the replicability and consistency of effects. *Replicability*, commonly defined as the ability to reproduce effects using the same methods but different data, was quantified in terms of the correlation between neural measures and pain intensity in the pooled replication sets, i.e., after merging the six replication sets into one. *Consistency* was quantified in terms of a consistency score, which is described in more detail in the Supplementary Materials. In the discovery + replication approach, ComBat-based feature harmonisation was performed separately within the discovery and replication sets.

Finally, mega-analyses in which the discovery and all replication sets were merged and analysed as one joint dataset were performed. In univariate mega-analyses, ComBat-based feature harmonisation was performed across all datasets.

### Machine learning analyses

We trained and tested ML models that relate multivariate patterns of brain connectivity across theta, alpha, and beta frequencies to the dependent variables of interest: pain intensity for primary analyses and age for a control analysis. The machine learning pipeline was implemented in MATLAB (R2021a, Statistics and Machine Learning Toolbox) using, at its core, the lasso function, which supports elastic net regression. The procedure for training and testing the ML models, inspired by the approaches in^9^ and,^8^ is described in the following.

To identify multivariate patterns of brain connectivity, we employed elastic net regression.^59^ This approach generates predictions as a weighted sum of model features. Here, these model features primarily correspond to connectivity values associated with different pairs of large-scale brain networks. The model weights are determined by minimising the mean square deviation between model predictions and observations while incorporating L1- and L2-norm regularisation terms. The objective function comprises hyper parameters λ>0 and α∈(0,1) which control the overall degree of regularisation and the relative influence of L1- and L2-regularisation, respectively. By introducing these regularisation terms, estimates of model weights become sparse (less important model weights are set to zero) and the model has the capacity to detect generalisable patterns in cases where the number of model features exceeds the number of observations. Moreover, elastic net regression is robust in settings with highly correlated features, such as EEG-based connectivity estimates.

We assessed model performance using both “in-sample” cross-validation (CV) and “out-of-sample” testing. For in-sample CV we employed two approaches: leave-one-participant-out cross-validation (LOO-CV) and leave-one-study-out cross-validation (LOSO-CV). In LOO-CV, a model was trained on all available data points except one. The fitted model was then used to predict the target value of the omitted (test) data point. This process was iterated, leaving out a different data point in each cycle, until all data points had been used as test data exactly once. Data standardisation (ComBat-harmonisation and z-scoring variables within studies) and confounder removal (regressing-out age effects) were conducted without allowing information leakage from test to training sets. Specifically, study-specific transformation functions and age effects were determined using solely the training set and subsequently applied in both the training and test sets. In LOSO-CV, data splits of each CV-fold were performed at the study level rather than the subject level. Moreover, data normalisation and confounder removal were performed independently for the training and test sets. A practical complication arises because ComBat-harmonisation can adjust only features from sites that it has seen during training. However, LOSO-CV implies that site identifiers occurring in the test set do not occur in the training set. To address this, we followed the approach described in^38^: For each participant in the test set, we generated a separate prediction for each site identifier present in the training set, each time assuming the participant originated from that site. The resulting site-specific predictions were then averaged, yielding a single estimate per individual in the test set. For both LOO-CV and LOSO-CV, we statistically assessed the in-sample model performance by computing Bayes factors determining the evidence for a positive correlation between all predicted and observed target values.

In each CV-fold, optimal values for hyper parameters λ and α were determined by comparing associated prediction-outcome correlations estimated using a nested CV-loop within the training set. For α, we explored values [0.01, 0.02, 0.04, 0.08, 0.16, 0.32, 0.64]. For λ we considered 20 different values with a ratio of 0.001 between smallest and largest value. The largest value for λ was adapted dynamically for each value of α such that it gave rise to a model with only zero coefficients. Thus, in total, 140 different parameter settings were explored.

In out-of-sample testing, we trained a model on the discovery dataset and predicted observations in the standardised and confounder-corrected replication datasets. To statistically assess the out-of-sample model performance, we computed Bayes factors reflecting the evidence for a positive correlation between predicted and observed target values in the replications sets.

### Role of funders

This research was funded by the Deutsche Forschungsgemeinschaft (SFB1158, PL321/14-1) and the Technical University of Munich (TUM Innovation Network *Neurotech*). The funders did not have any role in the study design, data collection, data analyses, interpretation, or writing of the report.

## Results

### Standard analysis: how do standard EEG features relate to pain intensity?

**Fig. 4:**
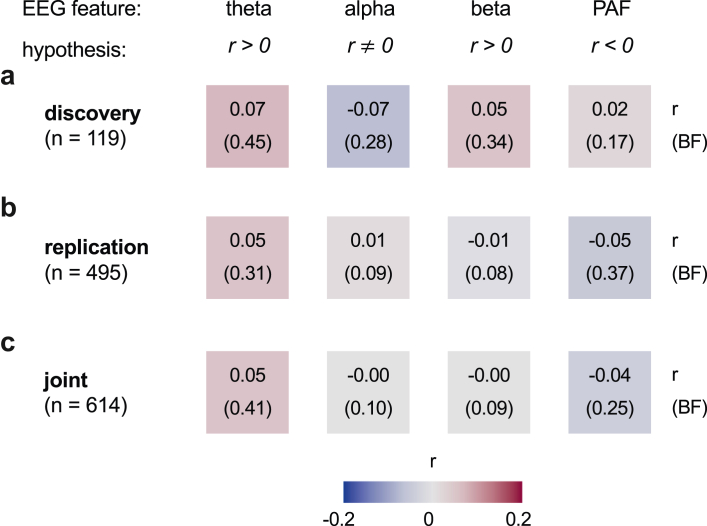
**Univariate correlations between standard EEG features and pain intensity.** (a) Correlations between pain intensity and EEG features in the discovery set. Each tile's top number and colour represent the correlation coefficient, and the bottom number is the associated BF. (b) Correlations in the pooled replication sets. The meanings of numbers and colours match those in panel (a). (c) Correlations in the joint set. The meanings of numbers and colours match those of panel (a).

First, we assessed how standard EEG features relate to chronic pain intensity. Chronic pain intensity was measured by ratings of the average pain intensity over the past one to four weeks (depending on the dataset). We opted for this pain measure because it was consistently available across all datasets. As standard EEG features, we assessed global absolute signal power at theta, alpha, and beta frequencies and the peak alpha frequency (PAF) since these features are the most frequently investigated in chronic pain.^4^ Based on prior evidence,^4^ we conducted one-sided tests to determine whether theta and beta power positively correlated and PAF negatively correlated with pain intensity. As no hypotheses about directionality existed for alpha power, we assessed its correlation with pain intensity bidirectionally. We found anecdotal to moderate evidence against correlations between pain intensity and all examined features in the discovery and replication sets (BF < 1 in the discovery set (Fig. 4a), BF < 1 in the replication sets (Fig. 4b)). Similarly, the mega-analysis yielded evidence against correlations between all tested EEG features and pain intensity (Fig. 4c). To assess the robustness of the lack of correlation between PAF and pain intensity, we repeated the analysis using the local maximum method for computing the PAF instead of the centre of gravity method. The results were consistent, showing no evidence for a correlation (discovery: r = 0.07, BF = 0.13; replication: r = 0.04, BF = 0.06; joint: r = 0.045, BF = 0.05). Thus, the largest EEG database on people with chronic pain to date provided evidence against a significant relationship between standard EEG features and chronic pain intensity.

### Network analysis: how does brain network connectivity relate to pain intensity?

Next, we investigated how the connectivity of large-scale brain networks relates to chronic pain intensity. This network analysis focused on connectivity between the seven large-scale brain networks defined by the Yeo atlas^11^ (Fig. S1a). We first extracted signals representative of individual networks using a newly developed and simulation-validated signal orthogonalisation algorithm (see Supplementary Materials). Subsequently, we computed the amplitude envelope correlation (AEC)^50^ between these representative signals. We used an amplitude-based connectivity metric as it is conceptually close to fMRI-based connectivity assessments on which the large-scale brain network concept is based.^11^ Connectivity values were extracted for three canonical frequency bands^60^: theta (3–8 Hz), alpha (8–13 Hz), and beta (13–30 Hz). This preregistered focus was motivated by prior evidence highlighting these bands' relevance in neuropsychiatric disorders and chronic pain.^4^^,^^10^^,^^61^ Gamma frequencies were excluded as this multi-site study incorporated data from various acquisition systems, where higher frequencies are likely more prone to systematic noise differences due to lower signal-to-noise ratio. Thus, in total, we computed 63 connectivity values per participant. We related these connectivity values to pain intensity using both univariate correlations and multivariate machine learning.

#### Univariate analyses

**Fig. 5:**
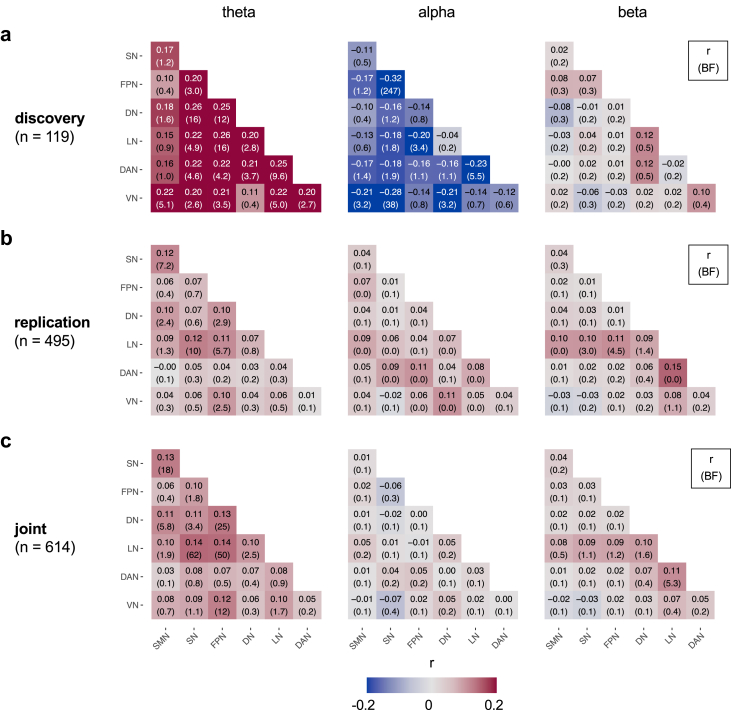
**Univariate correlations between pain intensity and brain network connectivity at theta, alpha, and beta frequencies.** (a) Correlations between pain intensity and brain network connectivity in the discovery set. Each heatmap tile's top number and colour represent the correlation coefficient; the bottom number is the associated BF. (b) Correlations in the pooled replication sets. The meanings of numbers and colours match those of panel (a). (c) Correlations in the joint set. The meanings of numbers and colours match those of panel (a). SMN, somatomotor network; SN, salience network; FPN, frontoparietal network; DN, default network; LN, limbic network; DAN, dorsal attention network; VN, visual network.

First, we sought to determine how connectivity of individual brain network pairs relates to pain intensity in the discovery set (Fig. 5a). In the discovery set, pain intensity correlated positively with all connectivity values at theta frequencies and correlated negatively with all connectivity values at alpha frequencies.

In the theta band, evidence for a correlation with pain intensity was at least anecdotal for 18 out of 21 network pairs and moderate to strong for 13 out of 21 network pairs (Fig. 5a, first panel). In the alpha band, evidence for such correlations was at least anecdotal for 13 out of 21 network pairs and moderate to strong in 6 out of 21 network pairs (Fig. 5a, second panel). In the beta band, we found moderate evidence against correlations between pain intensity and brain network connectivity (Fig. 5a, third panel).

Second, we assessed the replicability effects in the seven replication datasets (Fig. 5b). The results revealed that the replicability was generally low but markedly higher at theta than at alpha frequencies. In the theta band, in 6 out of 18 network pairs, which had shown at least anecdotal evidence for a correlation with pain intensity in the discovery set, there was at least anecdotal evidence for replicability. Three of these network pairs (SN-SMN, LN-SN, LN-FPN) showed moderate to strong evidence for replicability. By contrast, for all network pairs at alpha and beta frequencies showing at least anecdotal evidence for a correlation with pain intensity in the discovery set, we found evidence against replicability. We also evaluated a different measure of replicability, i.e., the consistency of effects across individual replication datasets, yielding qualitatively similar results (see Supplementary Materials and Fig. S2).

Third, we performed a mega-analysis on the joint dataset (Fig. 5c). The results provided strong evidence for a positive correlation between pain intensity and brain network connectivity at theta frequencies. Specifically, at theta frequencies, there was strong evidence for a positive correlation between pain intensity and brain network connectivity in 5 out of 21 network pairs and at least anecdotal evidence for such correlations in 12 out of 21 network pairs (Fig. 5c, first panel). Additionally, at beta frequencies, we found anecdotal to moderate evidence for a correlation with pain intensity in 4 out of 21 network pairs (Fig. 5c, second panel). At both theta and beta frequencies, the strongest correlations were observed for network pairs involving the Limbic network. At alpha frequencies, there was evidence against correlations between pain intensity and brain network connectivity (Fig. 5c, third panel) for all network pairs. By computing rank-based correlations and associated p-values in the joint set, we confirmed that associations between network connectivity and pain intensity were not driven by individual outliers (Fig. S2d). Analyses of gender-disaggregated data showed that, in both male and female subgroups the largest correlations with pain intensity occurred for network pairs at theta frequencies and involving the limbic network (Fig. S3).

We repeated the univariate analysis for the subgroup of individuals with chronic back pain (n = 405, Fig. S4). This analysis yielded qualitatively similar results to the primary analysis with all individuals.

#### Multivariate analyses

**Fig. 6:**
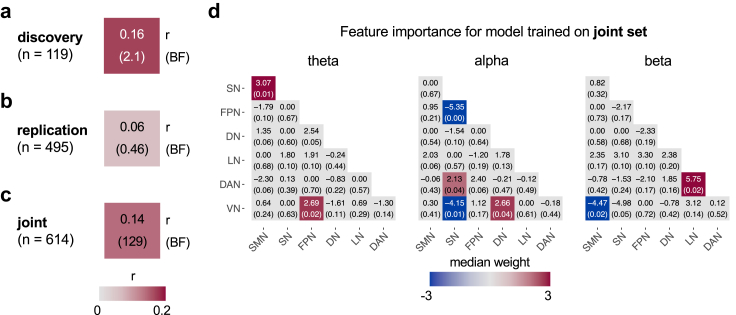
**Associations between pain intensity and multivariate patterns of brain network connectivity.** (a) In-sample, leave-one-participant-out cross-validated (LOO-CV) correlation between predicted and observed pain intensity in the discovery set. (b) Out-of-sample correlation between predicted and observed pain intensity in the pooled replication sets. (c) In-sample, LOO-CV correlation between predicted and observed pain intensity in the joint set. (d) Visualisation of corresponding model weights. The top number and colour of each tile represent the median of the weights across bootstrap samples. The bottom number represents the empirical p-value, i.e., the fraction of bootstrap samples for which the sign of this predictor differed from that of the median value. Only tiles with uncorrected empirical p < 0.05 are coloured.

Next, we investigated how multivariate patterns of brain network connectivity relate to pain intensity. To this end, we trained and tested machine learning (ML) models that employ connectivity values at theta, alpha, and beta frequencies as features. A model trained and tested on the discovery dataset yielded only anecdotal evidence for a cross-validated correlation between predicted and observed pain intensity (“in-sample” cross validation (CV), r = 0.16, BF = 2.1, Fig. 6a). When testing this model in the seven replication sets (“out-of-sample” validation, Fig. 6b), we found no evidence for a correlation between predicted and observed pain intensity (r = 0.06, BF = 0.52).

By contrast, in the multivariate mega-analysis (Fig. 6c), i.e., when training and testing a model on the joint set, we found strong evidence for a cross-validated correlation between predicted and observed pain intensity (r = 0.14, BF = 129). A bootstrapping analysis of model weights indicated that predictions were driven by brain network connectivity in all frequency bands (Fig. 6d), with mostly positive weights at theta frequencies and both positive and negative weights at alpha and beta frequencies. The model's capacity to generalise to independent datasets was corroborated by a leave-one-study-out cross validation (LOSO-CV), yielding strong evidence for an association between predicted and observed pain intensity (r = 0.12, BF = 19). In LOSO-CV, the model is trained on data from all studies but one, and the prediction performance is assessed using the data from the left-out study. Prediction-observation correlations for each dataset based on LOSO- and LOO-CV are provided in Table S2.

We also trained and tested models using only the subgroup of individuals with chronic back pain (n = 405, Fig. S5). The subgroup models trained and tested on the discovery/joint set yielded qualitatively similar patterns. Still, evidence was weaker (no/anecdotal evidence for a cross-validated correlation between predicted and observed pain intensity in the discovery/joint set).

Next, we aimed to check the data quality and our methodology's sensitivity by replacing the dependent variable pain intensity with age, which is known to affect many measures of brain function.^39^^,^^40^^,^^62^ An ML model trained and tested on the discovery dataset predicted age significantly better than chance (Fig. S6a, r = 0.36, BF > 10^4^), and we found strong evidence for the replicability of its predictions in independent datasets (Fig. S6b, r = 0.17, BF = 319). Likewise, a model trained and tested in the joint set yielded strong evidence for a relationship between multivariate brain network connectivity patterns and age (Fig. S6c, r = 0.23, BF > 10^6^). These findings demonstrate that the data quality and the sensitivity of the methodology are sufficient to detect associations between brain network patterns and demographic variables.

#### Summary

Univariate analyses of the discovery set showed positive correlations between pain intensity and brain network connectivity at theta frequencies, and negative correlations between pain intensity and connectivity at alpha frequencies. The replicability of these results was rather low but higher at theta than at alpha frequencies. In a mega-analysis, an ML model trained and tested on the joint set yielded strong evidence for a pain intensity-related connectivity pattern. The cross-validated correlation between predicted and observed pain intensity was r = 0.14, corresponding to an explained variance of approximately 2%. Predictions of this model were driven by network features in all frequency bands.

### Explanatory analysis: how do other network features relate to pain intensity?

**Fig. 7:**
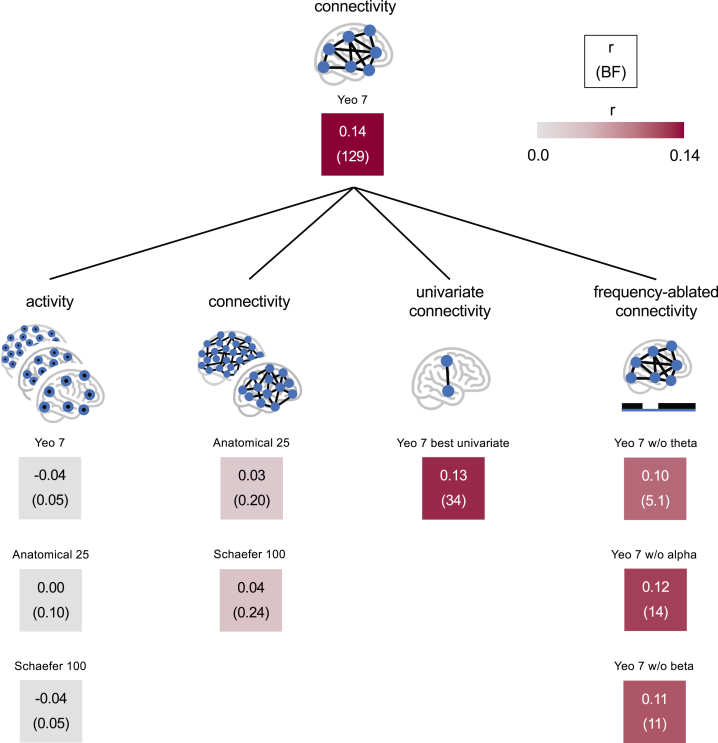
**Associations between pain intensity and several multivariate patterns of brain network features.** Each tile's top number and colour represent the leave-one-participant-out cross-validated correlation between predicted and observed pain intensity in the joint set. The top-centre tile shows the prediction-observation correlation for the model employing brain network connectivity features among the seven Yeo networks at theta, alpha, and beta frequencies as features. Bottom tiles show the prediction-observation correlations of models employing alternative network properties as features.

The multivariate network model, trained and tested in the joint dataset, yielded strong evidence for a cross-validated correlation between predicted and observed pain intensity ratings (r = 0.14, BF = 129). To better understand which model components contribute most to its predictions, we compared its prediction accuracy to that of various alternative models that used different network properties as features (Fig. 7).

We considered the following alternative models and compared their performance to the reference network model by computing BF-ratios (BF-ratio = BF of reference model/BF of alternative model). First, a model using brain network activity instead of brain network connectivity of the 7 Yeo networks yielded evidence against a positive correlation between predicted and observed pain intensity (BF-ratio > 10^3^), indicating the higher information content concerning pain intensity of brain network connectivity compared to brain network activity. This result did not change when considering patterns of brain activity in 25 anatomically defined regions or in 100 relatively fine-grained brain parcels defined by the Schaefer atlas.^56^ Second, models using brain connectivity among 25 anatomically defined brain regions or among 100 relatively fine-grained brain parcels defined by the Schaefer atlas^56^ (Fig. S1b and c) showed evidence against a positive prediction-outcome correlation (BF-ratio > 537), indicating that information about pain intensity is more efficiently extracted on the level of large-scale functional networks than on the level of fine-grained brain regions or parcels. Third, we tested whether multivariate patterns of brain network connectivity capture pain intensity-related information better than individual network features. To this end, we reassessed the individual network feature that previously showed the strongest correlation with pain intensity (LN-SN connectivity in the theta band: r = 0.14, BF = 82) using the same cross-validation procedure used for the multivariate model. While the evidence for the cross-validated univariate correlation was strong (r = 0.13, BF = 34), it was still substantially weaker than that of the multivariate model (BF-ratio = 3.8). This suggests that complex multivariate patterns of brain network connectivity represent pain intensity-related information more effectively than connectivity of individual network pairs. Next, we evaluated the information content of individual frequency bands by training and testing models that excluded network features from the theta, alpha, or beta frequency bands. All frequency-ablated models provided at least moderate evidence for a positive prediction-observation correlation. However, the substantially weaker evidence in the frequency-ablated models compared to the model employing connectivity features of all three frequency bands (BF-ratio > 9.2) suggests that all three frequency bands contain relevant information about pain intensity.

In addition to varying feature types described above, we also varied the method used to compute them. Specifically, we tested whether our newly developed algorithm for identifying representative brain network signals preserved more pain intensity-related information than a standard PCA-based approach. A model using PCA-derived connectivity features showed a reduced prediction-outcome correlation compared to the reference network model (BF-ratio = 43), indicating that our algorithm for representative signal identification can enhance the pain intensity-related information content of network connectivity features.

## Discussion

Characterising the brain mechanisms of chronic pain and, on this basis, developing brain-based biomarkers, is a key challenge in pain research. In this study, we investigated the relationship between chronic pain intensity and various EEG features. We scrutinised the replicability and consistency of these associations by analysing eight independent datasets, resulting in the largest EEG data analysis in people with chronic pain so far. Our analyses of standard EEG features provided robust evidence against associations with pain intensity. Building on findings from previous neuroimaging studies, we next analysed connectivity between the seven canonical large-scale brain networks defined by the Yeo atlas.^11^ Employing a discovery-replication approach, we found that associations identified in the discovery dataset were inconsistently replicated in other datasets. A mega-analysis combining all datasets revealed the most robust associations between pain intensity and connectivity across large-scale brain networks at theta frequencies and specifically in network pairs involving the LN. Additionally, multivariate analyses identified connectivity patterns spanning theta, alpha, and beta frequencies exhibiting strong evidence for associations with pain intensity. Variations and ablations of model features yielded deeper insights into the relative importance of distinct electrophysiological brain features in assessing chronic pain, providing guidance for developing EEG-based, scalable, and affordable biomarkers of chronic pain.

### Standard analysis

As a first step, we assessed how pain intensity related to commonly analysed EEG features, i.e., frequency band-specific power and peak alpha frequency. A recent systematic review reported enhanced theta and beta power and a reduced peak alpha frequency in resting-state M/EEG recordings of people with chronic pain.^4^ Our analyses mainly provided evidence against correlations between these EEG features and chronic pain intensity. These findings do not rule out associations between chronic pain and commonly analysed EEG features but show that these associations are weaker and less robust than sometimes assumed. Intriguingly, they were weaker than associations between chronic pain and the brain network connectivity features considered here.

### Network analysis

In the discovery dataset, we found strong evidence for associations between pain intensity and brain network connectivity in numerous network pairs at theta and alpha frequencies. Evidence for the replicability of these associations was found at theta frequencies but not at alpha frequencies. A mega-analysis of the combined discovery and replication sets corroborated the association between pain intensity and brain network connectivity at theta frequencies, specifically in network pairs involving the limbic network. These findings have two key implications: First, they reveal an association between chronic pain and brain network connectivity at theta frequencies. Second, they highlight that strong evidence for an association in the discovery dataset does not necessarily imply replication success. Therefore, future studies investigating associations between brain features and clinical variables should prioritise replication in independent datasets. This is particularly important when the variability of recording conditions in discovery datasets is lower than in replication datasets. In addition to these main findings, our results indicate that connections involving the limbic network exhibited the strongest associations with pain intensity. While EEG has limited spatial resolution, especially for deep structures, this pattern is consistent with previous fMRI studies implicating altered limbic, salience, and default mode network function in chronic pain.^63^ The limbic system plays a central role in the emotional and motivational modulation of pain, including the assignment of salience to sensory inputs and regulation of affective responses. These findings therefore align with the notion that affective-motivational processes are core features of persistent pain.

To evaluate the relationship between pain intensity and multivariate patterns of brain network connectivity, we trained and tested ML models using brain network connectivity features at theta, alpha, and beta frequencies. A model trained on the discovery set showed only anecdotal evidence for a positive cross-validated prediction-observation correlation and did not generalise to independent data. In contrast, a model trained and tested on the joint dataset yielded strong evidence for a positive prediction-observation correlation, indicating multivariate pain intensity-related patterns of brain network connectivity. By successfully relating brain network connectivity to pain intensity in a large and heterogeneous dataset, our model serves as a benchmark and methodological guide for future EEG-based prediction models of pain intensity in people with chronic pain.

### Explanatory analysis

Having identified a multivariate model with strong evidence for an association between brain network connectivity and pain intensity, we aimed to determine which aspects of the model enabled this association. This analysis sought to provide deeper insights into the types of EEG features most likely to contain chronic pain-related information, thereby guiding future efforts to discover EEG-based biomarkers for chronic pain. By comparing the prediction accuracy of our model to several alternative models that employed different types of network properties as features, we derived four key insights. First, brain network connectivity is more informative about pain intensity than brain network activity. Second, brain network connectivity on the level of large-scale functional networks is more informative about pain intensity than connectivity on the level of fine-grained brain parcels. Third, multivariate patterns of brain network connectivity are more informative about pain intensity than the connectivity of individual network pairs. Fourth, brain network connectivity features across all considered frequencies, i.e., at theta, alpha, and beta frequencies, provide complementary information about pain intensity. Together, these insights suggest that future studies aiming to identify EEG-based correlates of chronic pain might focus on multivariate connectivity patterns across large-scale brain networks at multiple frequency bands.

### Possible reasons for small effect sizes and low replicability

Our analyses revealed associations between pain intensity and brain network connectivity. However, the effect sizes were small, and the replicability was limited. Several explanations could account for these findings:

First, EEG signals may not contain sufficient information about pain intensity to give rise to larger and more replicable effects. Considering the converging evidence for changes in cortical function in chronic pain states in animals and humans,^4^^,^^6^^,^^23^^,^^24^^,^^41^^,^^64^, ^65^, ^66^, ^67^, ^68^ this explanation is unlikely.

Second, EEG signals may contain substantial information about pain intensity, but the current measures of brain connectivity may not extract this information efficiently. Considering the crucial role of connectivity in shaping pain from fMRI and EEG studies in both human and animal studies,^5^^,^^8^^,^^9^^,^^69^^,^^70^ this explanation also appears unlikely.

Third, EEG-based connectivity contains substantial information about pain intensity, but the current approach to estimating brain network connectivity is suboptimal. In light of the multiverse of possible connectivity analyses, we deem this explanation possible. Although we derived all analytical choices from theoretical and empirical considerations, a different connectivity measure (e.g., phase- instead of amplitude-based), a different source reconstruction algorithm (e.g., minimum norm instead of beamforming), a different approach for computing band-specific signal envelopes (e.g., wavelets rather than Hilbert transform), different definitions of frequency bands (e.g., subdividing canonical bands into narrower sub-bands), or simply a different epoch length might have been better choices. Future studies might systematically explore the multiverse of connectivity analyses to find the most informative approach.

Fourth, the current approach to EEG connectivity is appropriate, but the data are too heterogeneous and noisy. This is plausible given that our cohort included individuals of different genders and encompassed diverse types of chronic pain, such as chronic back pain, chronic widespread pain, joint pain, and neuropathic pain. These different chronic pain types are shaped to varying degrees by nociceptive, neuropathic, and nociplastic components which likely involve distinct brain mechanisms. In the trade-off between sample size and homogeneity and given the available datasets, we opted for a large but heterogeneous sample of people with chronic pain in our primary analyses. To test whether stronger patterns would emerge for a more homogeneous group, we conducted a control analysis in the subgroup of people with chronic back pain. This analysis, however, yielded qualitatively similar results to the full sample. Nevertheless, even chronic back pain represents a mixed pain condition, influenced by different combinations of nociceptive, neuropathic, and nociplastic factors. Thus, to achieve true homogeneity, future studies might focus on large cohorts of people with chronic pain with more homogeneous profiles of nociceptive, neuropathic, and nociplastic components. Beyond, the present study's EEG recording conditions (e.g., EEG devices or room temperature) and clinical assessments (e.g., number and type of presented questionnaires or investigator characteristics) were also heterogeneous. To mitigate these differences, we harmonised EEG features across sites with the dedicated ComBat batch-effect correction method and removed site-level variability from dependent variables by converting them to within-site z-scores. Having eliminated all site-level variability in the dependent variables, we consider the present approach maximally conservative. In our multivariate analysis, we further performed leave-one-study-out cross-validation, a validation strategy inherently robust to spurious, non-physiological site effects. Still, systematic differences in data quality, may have led to a scenario where effects occur in some data sets but not in others, thereby attenuating overall effect sizes. Because no validated measures for EEG data quality exist, we did, however, not exclude datasets based on potential data quality issues. In short, despite multiple steps to reduce dataset heterogeneity, it likely remains substantial, complicating the detection of larger effects. Multi-centre studies going forward should therefore further reduce data heterogeneity at the acquisition stage by standardising EEG recording conditions and clinical assessments.

Fifth, while the replication data might be too heterogeneous, one could argue that the discovery data were too homogeneous. To identify robust and generalisable effects, future studies should aim to discover data encompassing the heterogeneity of potential replication datasets. Moreover, discovery datasets must be large to effectively identify effects in the presence of data heterogeneity. Large datasets with realistic degrees of heterogeneity may be curated by combining data from diverse origins, ideally achieved via collaborative initiatives.

### Conclusions and future directions

In the present study, we investigated associations between various EEG features and pain intensity in people with chronic pain and assessed the replicability of effects across eight independent datasets. Employing a discovery-replication approach, we found limited replicability of associations between pain intensity and brain network connectivity. A mega-analysis combining all datasets revealed the most robust associations between pain intensity and connectivity between large-scale brain networks at theta frequencies. Additionally, multivariate analyses identified connectivity patterns spanning theta, alpha, and beta frequencies that exhibited strong evidence for associations with pain intensity. Further analyses confirmed that the multivariate assessment of connectivity across large-scale brain networks is more informative about chronic pain than standard EEG features assessed in previous studies. Together, these findings highlight the potential of brain connectivity patterns rather than standard EEG features to serve as biomarkers of chronic pain. Moreover, they underscore the importance of assessing replicability in independent data. Future research might thus investigate connectivity patterns in collaborative, multi-centre studies, such as those coordinated by initiatives like ENIGMA-Chronic Pain.^71^^,^^72^ Ideally, such studies should include large pre-defined sample sizes, homogenous types of chronic pain, and standardised EEG and clinical assessments. The methodology developed in this study offers a blueprint for such investigations. In this way, future research promises novel insights into the brain mechanisms of chronic pain, aiding the development of clinically valuable biomarkers and ultimately improving the individualised treatment of chronic pain.

## Contributors

All authors have read and approved the final version of the manuscript.

Felix S. Bott: Conceptualisation, Methodology, Formal analysis, Investigation, Data curation, Writing – Original Draft, Writing – Review & Editing, Visualisation.

Paul Theo Zebhauser: Investigation, Resources, Writing – Review & Editing.

Vanessa D. Hohn: Investigation, Resources, Writing – Review & Editing.

Özgün Turgut: Methodology.

Elisabeth S. May: Investigation, Resources, Writing – Review & Editing, Data curation.

Laura Tiemann: Investigation, Resources, Writing – Review & Editing.

Cristina Gil Ávila: Resources, Data curation.

Henrik Heitmann: Investigation, Resources.

Moritz M. Nickel: Investigation, Resources.

Melissa A. Day: Investigation, Resources, Writing – Review & Editing.

Divya B. Adhia: Investigation, Resources, Writing – Review & Editing.

Yoni K. Ashar: Investigation, Resources, Writing – Review & Editing.

Tor D. Wager: Investigation, Resources, Writing – Review & Editing.

Yelena Granovsky: Investigation, Resources.

David Yarnitsky: Investigation, Resources, Writing – Review & Editing.

Mark P. Jensen: Investigation, Resources, Writing – Review & Editing.

Joachim Gross: Conceptualisation, Methodology, Writing – Review & Editing.

Markus Ploner: Conceptualisation, Resources, Writing – Original Draft, Writing – Review & Editing, Visualisation, Supervision, Project Administration, Funding acquisition.

## Data sharing statement

The EEG and meta data for the datasets Set_Brisbane, Set_Otago, Set_Otago2, Set_Boulder, Set_Haifa, and Set_Seattle are not deposited in a public repository due to formal data sharing agreements with the collaborating research institutions. The EEG and meta data of dataset Set_Munich1 are publicly available at https://osf.io/srpbg/. The EEG and meta data of dataset Set_Munich2 cannot yet be deposited in a public repository as they are part of an ongoing study. Public access to Set_Munich2 will be provided upon completion and publication of the study at https://osf.io/mj9xr/. The code is available at https://osf.io/4qmyw/. Any additional information required to reanalyse the data reported in this paper is available from the lead contact upon request.

## Declaration of generative AI and AI-assisted technologies in the writing process

During the preparation of this work the authors used chatGPT 4o in order to improve the readability and language of this manuscript. After using this tool, the authors reviewed and edited the content as needed and take full responsibility for the content of the published article.

## Declaration of interests

YKA received consulting fees from Pain Reprocessing Therapy Center and has stocks in Lin Health. TDW is on the NCCIH Data Safety and Monitoring Board as well as on the Scientific advisory board of Curable Health.
