# Supplementary material for: Exploring electroencephalographic chronic pain biomarkers: a mega-analysis
Source: eBioMedicine. 2025 Sep 30;120:105955. doi: 10.1016/j.ebiom.2025.105955 (PMC12517086; doi:10.1016/j.ebiom.2025.105955)
Supplement: Supplementary Materials [file mmc1.docx]

# Supplementary materials

### Overview

- Participants table with gender information
- Network definitions
- Univariate analysis with consistency score and Spearman’s $\rho$
- Univariate mega analysis for gender-disaggregated data
- Multivariate analysis: Model performance in individual datasets.
- Univariate analysis in CBP subgroup
- Multivariate analysis in CBP subgroup
- Control analysis: Does network connectivity relate to age?
- Consistency score
- Simulative assessment of representative time series method

### Participants table with gender information

|  | dataset name | N_192_ | N_CBP_ | N_CWP_ | N_JP_ | N_NP_ | N_OTHER_ | #sens |
| --- | --- | --- | --- | --- | --- | --- | --- | --- |
| **disc. set** | *Set_Munich1*  *Set_Munich1 (f)* | 119  77 | 74  41 | 13  12 | 12  9 | 20  15 | 0  0 | 64 |
| **Repl. sets** | *Set_Munich2*  *Set_Munich2 (f)* | 63  36 | 21  13 | 2  2 | 11  6 | 17  7 | 12  8 | 32 |
|  | *Set_Brisbane*  *Set_Brisbane (f)* | 60  33 | 60  33 | 0  0 | 0  0 | 0  0 | 0  0 | 64 |
|  | *Set_Otago1 ^(x)^*  *Set_Otago1 (f)* | 57  $\geq$39 | 57  $\geq$39 | 0  0 | 0  0 | 0  0 | 0  0 | 64 |
|  | *Set_Otago2* ^(^*^)^  *Set_Otago2 (f)* | 77  54 | 77  54 | 0  0 | 0  0 | 0  0 | 0  0 | 64 |
|  | *Set_Boulder* ^(^*^)^  *Set_Boulder (f)* | 68  29 | 68  29 | 0  0 | 0  0 | 0  0 | 0  0 | 19 |
|  | *Set_Haifa*  *Set_Haifa (f)* | 47  17 | 0  0 | 0  0 | 0  0 | 47  17 | 0  0 | 64 |
|  | *Set_Seattle*  *Set_Seattle (f)* | 123  N/A | 48  N/A | 0  0 | 0  0 | 0  0 | 75  N/A | 128 |
|  | *Total*  *Total (f)* | **614**  $\boldsymbol{\geq}$**285** | **405**  $\boldsymbol{\geq}$**209** | **15**  **14** | **23**  **15** | **84**  **39** | **87**  **> 8** |  |

**Table S1: Overview of datasets with gender information.** The study included eight datasets, each comprising resting-state EEG and metadata of people with different types of chronic pain. To ensure accurate EEG feature estimates, we included only participants with a minimum of 192 clean EEG data epochs (see methods section for details). Chronic back pain (CBP) was the most frequent type of chronic pain. Further types chronic pain included chronic widespread pain (CWP), joint pain (JP), neuropathic pain (NP), and miscellaneous types of chronic pain (OTHER). The top number in each row indicates the total number of participants, the bottom number indicates the number of participants with female gender. Sets marked with ^(^*^)^ one individual of diverse gender. Sets marked with *^(x)^* include one individual for which the gender is unknown. For *Set_Seattle* no gender information is available.

### Network definitions

**Figure S1:** Visualisation of different spatial configurations. (a) Axial, coronal, and sagittal views of the somatormotor (SMN), the salience ventral attention (SN), the frontoparietal (FPN), the default (DN), the limbic (LN), the dorsal attention (DAN), and the visual network (VN). In primary network analyses, we focused on only four out of these seven networks (indicated by the underlined network abbreviations). In extended network analyses, we included all seven networks. (b) Axial, coronal, and sagittal views of the 25 regions of the Anat-25 spatial configuration. For visualisation purposes, the Anat-25-regions are grouped by their Yeo network affiliation; that is, each Anat-25-region belongs to exactly one Yeo network. For example, two out of 25 regions correspond to the left and right halves of the SMN, another five out of 25 regions correspond to five subdomains of the SN, and so on. Different colors are used to distinguish the Anat-25-regions within each Yeo network (due to a limited palette, colors do not distinguish regions across different Yeo networks). (c) Axial, coronal, and sagittal view of the 100 parcel centroids of the Schaefer-100 spatial configuration.

### Univariate analysis with consistency score and Spearman’s $\rho$

###

**Figure S2: Univariate correlations between pain intensity and brain network connectivity at theta, alpha, and beta frequencies and pain intensity – including consistency score.** (a) Correlations between pain intensity and brain network connectivity in the discovery set. Each heatmap tile’s top number and color represent the correlation coefficient; the bottom number is the associated BF. (b) Summary of correlations observed in the replication sets. The top number and color of each heatmap tile represent the consistency score (see supplementary methods for details). The bottom number represents the number of replication datasets that correlate with BF > 3 in the same direction as in the discovery set. (c) Correlations in the joint set. The meanings of numbers and colors match those of panel (a). (d) Correlations between pain intensity and brain network connectivity in the discovery set. Each heatmap tile’s top number and colour represent the rank-based correlation coefficient (Spearman’s $\rho$); the bottom number is the associated, FDR-corrected p-value. FDR correction was applied across all connections and frequency bands. SMN, somatomotor network; SN, salience network; FPN, frontoparietal network; DN, default network; LN, limbic network; DAN, dorsal attention network; VN, visual network.

Univariate mega analysis for gender-disaggregated data

**

**Figure S3: Univariate correlations between pain intensity and brain network connectivity at theta, alpha, and beta frequencies and pain intensity for gender-disaggregated data.** Each heatmap tile’s top number and color represent the correlation coefficient; the bottom number is the associated BF. (a) Correlations in the joint set for the subset of participants with female gender. (b) Correlations in the joint set for the subset of participants with female gender. SMN, somatomotor network; SN, salience network; FPN, frontoparietal network; DN, default network; LN, limbic network; DAN, dorsal attention network; VN, visual network.

Multivariate analysis: Model performance in individual datasets

|  | LOSO-CV | | | LOO-CV | | |
| --- | --- | --- | --- | --- | --- | --- |
| dataset | **r**  $\boldsymbol{\rho}$ | **P_Pears._**  ***p_Spear._*** | **BF** | **r**  $\boldsymbol{\rho}$ | **P_Pears._**  **p_Spear._** | **BF** |
| Set_Otago1 | **0.20**  ***0.24*** | **0.064**  ***0.034*** | **1.6** | 0.17  *0.15* | 0.11  *0.13* | 1.1 |
| Set_Otago2 | -0.01  *-0.02* | 0.55  *0.56* | 0.23 | 0.04  *0.04* | 0.35  *0.36* | 0.35 |
| Set_Brisbane | 0.15  *0.15* | 0.13  *0.12* | 0.89 | 0.14  *0.20* | 0.14  *0.062* | 0.84 |
| Set_Seattle | **0.24**  ***0.23*** | **0.0040**  ***0.0044*** | **12** | **0.17**  ***0.20*** | **0.029**  ***0.012*** | **2.3** |
| Set_Munich1 | 0.12  *0.16* | 0.089  *0.041* | 0.91 | **0.18**  ***0.22*** | **0.026**  ***0.0088*** | **2.5** |
| Set_Boulder | **0.26**  ***0.23*** | **0.017**  ***0.028*** | **4.3** | **0.31**  ***0.32*** | **0.0052**  ***0.0036*** | **11** |
| Set_Haifa | 0.11  *0.04* | 0.23  *0.40* | 0.63 | 0.11  *0.00* | 0.22  *0.49* | 0.64 |
| Set_Munich2 | -0.08  *-0.02* | 0.74  *0.55* | 0.18 | -0.00  *0.05* | 0.51  *0.34* | 0.28 |
|  |  |  |  |  |  |  |
| Pooled data | **0.12**  ***0.13*** | **0.0014**  ***0.0013*** | **19** | **0.14**  **0.16** | **0.00014**  **2.4e-5** | **129** |

**Table S2: Leave-one-study-out (LOSO) and leave-one-participant-out (LOO) cross-validated prediction-outcome correlations in individual datasets and in the pooled data.** The table provides Pearson’s r and the corresponding p-values and BFs in the individual dataset as well as in the pooled data. To assess the impact of outliers on the correlations, Spearman’s $\rho$ and corresponding p-value are also provided.

### Univariate analysis in CBP subgroup

**Figure S4: Univariate correlations between pain intensity and brain network connectivity at theta, alpha, and beta frequencies in the chronic back pain cohort.** (a) Correlations between pain intensity and brain network connectivity in the discovery set. Each heatmap tile's top number and colour represent the correlation coefficient; the bottom number is the associated BF. (b) Upper panel row: Correlations in the pooled replication sets. The meanings of numbers and colours match those of panel (a). Lower panel row: Summary of correlations observed in the replication sets. The top number and colour of each heatmap tile represent the consistency score (see supplementary methods for details). As there is one fewer replication set in the subgroup analysis, the probabilities under the Null hypothesis for observing a consistency score of at least 1 and 2 change to p < 0.05 and p < 0.1, respectively. The bottom number represents the number of replication datasets that correlate with BF > 3 in the same direction as in the discovery set. (c) Correlations in the joint set. The meanings of numbers and colours match those of panel (a). SMN, somatormotor network; SN, salience network; FPN, frontoparietal network; DN, default network; LN, limbic network; DAN, dorsal attention network; VN, visual network.

### Multivariate analysis in CBP subgroup

**Figure S5: Associations between pain intensity and multivariate patterns of brain network connectivity in the chronic back pain cohort.** (a) In-sample, leave-one-participant-out cross-validated (LOO-CV) correlation between predicted and observed pain intensity in the discovery set. (b) Given the negative prediction-observation correlation in the discovery data, an out-of-sample correlation between predicted and observed pain intensity in the pooled replication sets was not computed and is, hence, not displayed. (c) In-sample, LOO-CV correlation between predicted and observed pain intensity in the joint set. (d) Visualisation of corresponding model weights. The top number and color of each tile represent the median of the weights across bootstrap samples. The bottom number represents the empirical p-value, i.e., the fraction of bootstrap samples for which the sign of this predictor differed from the that of the median value. Only tiles with uncorrected empirical p < 0.05 are coloured.

### Control analysis: Does network connectivity relate to age?

**Figure S6: Associations between age and multivariate patterns of brain network connectivity.** (a) In-sample, leave-one-participant-out cross-validated (LOO-CV) correlation between predicted and observed pain intensity in the discovery set. (b) Out-of-sample correlation between predicted and observed pain intensity in the pooled replication sets. (c) In-sample, LOO-CV correlation between predicted and observed pain intensity in the joint set. (d) Visualisation of corresponding model weights. The top number and color of each tile represent the median of the weights across bootstrap samples. The bottom number represents the empirical p-value, i.e., the fraction of bootstrap samples for which the sign of this predictor differed from the that of the median value. Only tiles with uncorrected empirical p < 0.05 are coloured.

### Consistency score

In replication sets, we assessed both the *replicability* and *consistency* of effects identified in the discovery set. Details on the quantification of *replicability* are provided in methods section of the main text. To quantify *consistency*, we defined a consistency score as follows:

consistency score = max(0, N_ConsistSign_ + N_BF>3_ – 5).

Here, N_ConsistSign_ represents the number of replication sets for which the correlation direction (positive or negative) matches that of the discovery set, and N_BF>3_ indicates the number of replication sets for which, additionally, correlations yield a BF > 3. By assigning equal weights to all replication sets with BF > 3, the score penalises scenarios where replicability relies heavily on only a few or even a single replication set. The significance of a given consistency score level was estimated by simulating uncorrelated datasets (100,000 random instances) and determining the fraction of cases for which the observed score equaled or exceeded a certain level (see Fig. S7 for more details). The estimated probabilities for observing a consistency score of at least 1, 2, or 3 were p < 0.11, < 0.05, and < 0.01, respectively. We refer to score values of 1, 2, and 3 as anecdotal, moderate, and strong evidence for consistency, respectively. In the subgroup analysis, which included only individuals with chronic back pain, there was one fewer replication set, and the probabilities associated with different consistency score values were adjusted. In this case, the estimated probabilities for observing a consistency score of at least 1, 2, or 3 were p < 0.05, < 0.01, and < 0.001, respectively

**Figure S7: Consistency score.** To assess how consistently effects of the discovery set could be replicated in independent datasets, we devised a consistency score based on the number of replication sets for which the correlation direction matched that of the discovery set (N_ConsistSign_), and the number of replication sets for which, additionally, correlations yielded a BF > 3 (N_BF>3_). (a) For each combination of N_ConsistSign_ and N_BF>3_, we first computed the probability of observing an event at least as extreme. The results indicate that, in the relevant ranges (i.e., N_ConsistSign_ large, N_BF>3_ small), the probabilities have approximately the same order of magnitude for combinations where N_ConsistSign_ + N_BF>3_ = constant. (b) We, thus, defined a consistency score proportional to N_ConsistSign_ + N_BF>3_. To link the score values to quantitative statements, we estimated the probabilities of observing at least a certain score value under the Null hypothesis. For the case of seven replication sets, the probabilities under the Null hypothesis of observing a score value of a least 1, 2, or 3 are p < 0.11, < 0.05, and < 0.01, respectively. For the case of six replication sets, which is relevant for the CBP subgroup analysis, the probabilities under the Null hypothesis of observing a score value of a least 1, 2, or 3 are p < 0.05, < 0.01, and < 0.001, respectively.

### Simulative assessment of representative signals method

Assessing connectivity between large-scale brain networks using EEG is challenging due to the complex and intertwined geometries of the individual networks. A widely used method for quantifying connectivity between two smaller and simply-shaped brain structures, such as parcels or regions, is to calculate the mean of the corresponding entries of a high-resolution connectivity matrix [1, 2]. However, when applied to entire large-scale brain networks, this approach yields connectivity values that are highly correlated across individuals (pilot assessments yielded average r-values > 0.97). This spatial indifference of connectivity values is more likely attributable to the low spatial resolution of EEG than to the true underlying network dynamics: At the low spatial resolution of EEG, a reconstructed signal at a brain source of interest is contaminated by contributions originating from surrounding sources. In particular for sources in more slender regions of a network, this means that the associated signals are contaminated by contributions from sources belonging to other networks. In other words, many signals associated with a particular network do not appropriately represent that network’s dynamics. Therefore, an alternative way to compute inter-network connectivity is to first identify signals that are believed to be sufficiently representative of the respective networks. Connectivity between networks is then determined by evaluating the connectivity between their representative signals [3]. As outlined in the main methods, we have developed a family of methods aiming to effectively extract signals representative of large-scale networks.

To determine the optimal strategy for computing representative time series, a simulation experiment was conducted. In this experiment, we simulated ground truth signals at 400 locations in the brain, denoted as $\mathbf{G}\in\mathbb{R}^{400\times n}$. Signals differed between all 400 sources, but signals of sources belonging to the same network possessed a common component, i.e.,

$$\begin{matrix} \mathbf{G}^{A}=c_{\text{mix}}\mathbf{G}^{A,\text{common}}+\left( 1-c_{\text{mix}} \right)\mathbf{G}^{A,\text{indi}}, \end{matrix}$$

where $\mathbf{G}^{A}$ refers to those rows of $\mathbf{G}$ corresponding to netA. The rows of $\mathbf{G}^{A,\text{common}}$ are identical, while the rows of $\mathbf{G}^{A,\text{indi}}$ are distinct. The parameter $c_{\text{mix}}$ controls the degree to which activity in a network is determined by the network-specific common component. To obtain the simulated source-reconstructed signals $\mathbf{S}\in\mathbb{R}^{400\times n}$, we applied a spatial blurring filter to the ground truth signals:

$$\begin{matrix} \mathbf{S}=\mathbf{MG}=\mathbf{F}^{\top}\left( \mathbf{F}\mathbf{F}^{\top}+\lambda\mathbf{I} \right)^{-1}\mathbf{FG} \end{matrix}$$

The so-called resolution matrix $\mathbf{M}\in\mathbb{R}^{400\times400}$ results from the composition of the inverse and forward models. The forward model is a linear mapping given by the lead field matrix $\mathbf{F}$ and the inverse model is a linear mapping given by the minimum norm spatial filter $\mathbf{F}^{\top}\left( \mathbf{F}\mathbf{F}^{\top}+\lambda\mathbf{I} \right)^{-1}$ with $\lambda=tr\left( \mathbf{F}\mathbf{F}^{\top} \right)/25$.

Ultimately, our objective was to compute the amplitude envelope correlation (AEC, [4]) between representative signals. For two signals $\mathbf{r}^{A}$ and $\mathbf{r}^{B}$, the AEC can be defined as

$$AEC\left( \mathbf{r}^{A},\mathbf{r}^{B} \right)=\frac{1}{2}\left[ corr\left( p\left( \mathbf{r}^{A} \right),p\left( \mathbf{r}^{B\perp A} \right) \right)+corr\left( p\left( \mathbf{r}^{B} \right),p\left( \mathbf{r}^{A\perp B} \right) \right) \right]$$

where $p\left( \mathbf{r} \right)$ denotes the envelope of $\mathbf{r}$. Further, $\mathbf{r}^{B\perp A}$ refers to the signal $\mathbf{r}^{B}$ phase-orthogonalised w.r.t. $\mathbf{r}^{A}$ and $\mathbf{r}^{A\perp B}$ refers to the signal $\mathbf{r}^{A}$ phase-orthogonalised w.r.t. $\mathbf{r}^{B}$. Hence, there are four signals involved in the computation of the AEC: $\mathbf{r}^{A}$, $\mathbf{r}^{B}$, $\mathbf{r}^{A\perp B}$, and $\mathbf{r}^{B\perp A}$.

To quantitatively compare the different methods for estimating representative time series, we computed both the minimum and average of the following values:

- $\nu\left( \mathbf{r}^{A},\mathbf{G}^{A,\text{common}} \right)$ = "fraction of variance of $\mathbf{G}^{A,\text{common}}$ explained by $\mathbf{r}^{A}$"
- $\nu\left( \mathbf{r}^{A\perp B},\mathbf{G}^{A,\text{common}} \right)$
- $\nu\left( \mathbf{r}^{B},\mathbf{G}^{B,\text{common}} \right)$
- $\nu\left( \mathbf{r}^{B\perp A},\mathbf{G}^{B,\text{common}} \right)$

Henceforth, the minimum and average of these four values are referred to as minimum and average explained variance scores, respectively.

**Figure S8:** Relative explained variance scores achieved by the different methods for the Yeo-7 spatial configuration. (a), (b) Results for the case $c_{\text{mix}}=0.5$ and for 64 random repetitions of the numerical experiment. Black circles indicate the mean of scores across repetitions. (c), (d) Averaged explained variance scores across repetitions for three values of the mixing parameter $c_{\text{mix}}$.

We computed the explained variance scores for mixing parameter values $c_{\text{mix}} \in\{0.1, 0.5, 0.9\}$. Fig. S8 shows the explained variance scores of the different methods averaged across all network pairs and for 64 random repetitions of the simulation experiment. The values presented in the figure are relative scores, defined as the ratio between the score obtained from the method of interest and the score obtained from the standard method, i.e., standard PCA without any orthogonalisation. The values in Fig. S8a and Fig. S8b correspond to the case $c_{\text{mix}}=0.5$. Scores averaged across repetitions for all tested values of $c_{\text{mix}}$ are provided in Fig. S8c and Fig. S8d.

The results demonstrate that both *pairwise* and *global* orthogonalisation can significantly enhance the variance of the *common* ground truth signal that is explained by the representative time series. Among the methods tested, the best performing method is the global orthogonalisation with $N_{c}=5$. This method exhibits an average improvement in the average and minimum explained variance scores of roughly 20% and 50%, respectively. The variant of this method with $N_{c}=3$ performs similarly well but offers the practically important advantage of being computationally less demanding. Furthermore, the pairwise orthogonalisation entails a notable improvement, as well, with an increase in average and minimum explained variance scores of roughly 7% and 38%, respectively.

We conclude that the primary method for extracting representative time series should be *global orthogonalisation* with $N_{c}=3$.

# References

1. Toll, R.T., et al., *An Electroencephalography Connectomic Profile of Posttraumatic Stress Disorder.* Am J Psychiatry, 2020. **177**(3): p. 233-243. doi: 10.1176/appi.ajp.2019.18080911

2. Zhang, Y., et al., *Identification of psychiatric disorder subtypes from functional connectivity patterns in resting-state electroencephalography.* Nat Biomed Eng, 2021. **5**: p. 309-323. doi: 10.1038/s41551-020-00614-8

3. Pellegrini, F., et al., *Identifying good practices for detecting inter-regional linear functional connectivity from EEG.* NeuroImage, 2023. **277**: p. 120218. doi: 10.1016/j.neuroimage.2023.120218

4. Hipp, J.F., et al., *Large-scale cortical correlation structure of spontaneous oscillatory activity.* Nat Neurosci, 2012. **15**(6): p. 884-90. doi: 10.1038/nn.3101
